# Challenges in supplying empirical proof for predictions derived from Species Distribution Models (SDMs): the case of an invasive cyanobacterium

**DOI:** 10.1038/s43705-023-00264-2

**Published:** 2023-06-06

**Authors:** Carlotta Meriggi, Maliheh Mehrshad, Richard K. Johnson, Ane T. Laugen, Stina Drakare

**Affiliations:** 1 Department of Aquatic Sciences and Assessment, Swedish University of Agricultural Sciences, Uppsala, 750 07 Sweden; 2 Department of Natural Sciences, University of Agder, Kristiansand, Norway

**Keywords:** Freshwater ecology, Limnology

## Abstract

Species distribution models (SDMs) calibrated with bioclimatic variables revealed a high probability for range expansion of the invasive toxin producing cyanobacterium, *Raphidiopsis raciborskii* to Sweden, where no reports of its presence have hitherto been recorded. While predictions focused on the importance of climate variables for possible invasion, other barriers to dispersal and successful colonization need to be overcome by the species for successful invasion. In this study, we combine field-based surveys of *R. raciborskii* (microscopy and molecular analysis using species-specific primers) of 11 Swedish lakes and in-silico screening of environmental DNA using 153 metagenomic datasets from lakes across Europe to validate the SDMs prediction. Field-based studies in lakes with high/low predicted probability of occurrence did not detect the presence of *R. raciborskii*, and in-silico screening only detected hints of its presence in 5 metagenomes from lakes with probability ranging from 0.059 to 0.825. The inconsistencies between SDMs results and both field-based/in-silico monitoring could be due to either sensitivity of monitoring approaches in detecting early invasions or uncertainties in SDMs that focused solely on climate drivers. However, results highlight the necessity of proactive monitoring with high temporal and spatial frequency.

Invasion of microorganisms to a new ecosystem usually becomes noticeable only after crucial ecosystem services have been jeopardized [1]. However, the invasion of species capable of toxin production to new areas demands vigilant and proactive surveillance. The invasive *Raphidiopsis raciborskii* is an example of a toxin-producing, nitrogen-fixing, and bloom-forming filamentous cyanobacterium [2]. Strains of *R. raciborskii* differ in their ability to produce (cyano)toxins (cylindrospermopsin and saxitoxin), known to affect cattle, wild animals and humans, as well as many ecosystem services such as drinking and recreational water resources [3–5]. *R. raciborskii*, a species of tropical origin, is currently expanding its range across Europe’s freshwater ecosystems [6–8]. As early detection of an invasive species is requisite for implementation of efficient management actions, identifying areas at risk of invasion is therefore of high priority [2]. For this purpose, predictive models are useful for assessing the suitable habitats for colonization. Species distribution models (SDMs) predictions based on bioclimatic factors can be used to complement patchy species distributions derived from sporadic samplings and occasional reports of presence/absence of a target species [9]. However, SDMs results should mainly be considered as early warnings, underpinning monitoring efforts rather than proof of presence/invasion [10]. SDMs are statistical procedures that link occurrence records of a species to environmental variables to estimate spatial distribution patterns using a correlative approach [11, 12], however, successful colonization also requires the dispersal and establishment of invasive species in the new ecosystem [13, 14]. That is why most such modeling efforts face the same argument of whether their predictions have been empirically supported bringing another challenge regarding the reliability in the early detection of invasive species in areas at high risk of being invaded.

In a previous study [15], we based the SDMs on published observations of *R. raciborskii* and environmental predictors obtained from climatic models to visualize and predict potential new habitats for *R. raciborskii* in Europe. While this species has not been reported in Sweden, our SDMs prediction revealed potential areas for range expansion in the southern and central regions of Sweden [15]. Here, we integrate field-based surveys in Sweden and in-silico screening of environmental DNA from lakes across Europe to validate the SDMs prediction and highlight challenges in supplying such empirical proofs.

To provide empirical proof for the potential expansion of *R. raciborskii* to Sweden, we selected a number of eutrophic shallow lakes, sampled in late summer, with high (>0.5) and low (<0.5) predicted probability of presence (Table 1) and performed microscopic and molecular surveys (Supplementary Information S.1., for detailed sampling methodology). Water and sediment samples were used for DNA extraction and the rpoC1 gene [16] was targeted and amplified with *R. raciborskii* specific primers cyl2/cyl4 [16] and cyl4F/cyl4R [17] (Supplementary Information S.1.3). The specific primers were tested on a European strain of *R. raciborskii* (NIVA-CYA 399, Norwegian Culture Collection of Algae) isolated from Lake Balaton (Hungary), this strain was also used as a positive control. The products of the species-specific polymerase chain reactions were separated by electrophoresis on 1.5% agarose gel and visualized under UV illumination. None of these 11 lakes resulted in the amplification of the target region suggesting the absence of *R. raciborskii*. However, since molecular methods could suffer from a limited detection range, the sensitivity and the detection limits of the method were evaluated. Accordingly, a total of 50, 100, and 500 filaments of the reference culture of *R. raciborskii* (NIVA-CYA 399) were picked using an inverted light microscope, and the same procedure used for field samples was followed. While the cyl4F/cyl4R returned a band for all three reactions, the cyl2/cyl4 primer was only able to return a band for the reactions with 100 and 500 filaments (Supplementary Fig. S1). This highlights the partial limitation of this molecular method in detecting the presence of this invasive species, especially during early stages of invasion when population densities are likely low. Using other molecular methods such as duplex digital PCR (dPCR) is reported to improve the detection limit [18]; however, requirements of such methods might not be as widely accessible as PCR. The negative results of molecular analyses were corroborated by the lack of microscopic identification of *R. raciborskii* in the samples (Supplementary Information, S.1.2). To complement the field study, in-silico screening of environmental DNA using publicly available lake metagenomes was also performed. A total of 153 metagenomic datasets from 50 lakes across Europe were selected from publicly available datasets stored in the National Center for Biotechnology Information (https://www.ncbi.nlm.nih.gov/) (Table 1). The 16 S rRNA reads were extracted from these metagenomes using SSU-align tool [19] and their taxonomy was assigned using BLAST [20] against Silva SSU 138.1 [21] (Supplementary Information, S.1.4). The probability of occurrence of each site based on the SDMs prediction covered probabilities from 0.055 to 0.846 (Table 1) with a median of 0.276 indicating that metagenome availability and selection was slightly biased towards sites which may not favor *R. raciborskii* settlement/survival. Only 5 out of 153 screened metagenomes contained reads matching the *R. raciborskii* 16 S rRNA sequence (Table 1 and Fig. 1). The lakes from which these 5 metagenomes originate are situated in areas with high probability of occurrence in three cases (0.537 to 0.825) and lower probability (0.059 and 0.319) in two cases. The low number of reads matching the *R. raciborskii* 16 S rRNA sequence makes it difficult to define a threshold in interpreting the SDMs prediction. In addition, lower abundances in the early stages of invasion poses limitations for in-silico methods in general and specifically for *R. raciborskii* since cyanobacteria are usually underrepresented in metagenomic datasets. Additionally, timing and frequency of sampling will also affect the efficiency of early detection methods as seen in the case of Rimov reservoir (predicted probability of 0.319), where only one of 38 metagenomes had a positive match (Table 1).

**Table 1:** Detected presence (+) and absence (−) of the invasive cyanobacterium *Raphidiopsis raciborskii* in European lakes using field-based or in-silico screening methods.

Country	Lake name	Accession (SRR/ERR)	Detection	Longitude	Latitude	Probability of occurrence	MetaG/ Field
Switzerland	Zurich	SRR12667570	−	8.5937	47.2832	0.095	MetaG
	Zurich	SRR12667427	−	8.5937	47.2832	0.095	MetaG
	Zurich	SRR12667319	−	8.5937	47.2832	0.095	MetaG
	Zurich	SRR11849211	−	8.5937	47.2832	0.095	MetaG
	Zurich	SRR11848270	−	8.6747	47.3519	0.106	MetaG
	Zurich	SRR7054681	−	47.3	8.57	0.104	MetaG
	Zurich	SRR6475630	−	47.3	8.57	0.104	MetaG
	Zurich	SRR6475632	−	47.3	8.57	0.104	MetaG
	Zurich	SRR6475633	−	47.3	8.57	0.104	MetaG
	Greifen	SRR11848494	−	8.6747	47.3519	0.106	MetaG
	Greifen	SRR11848491	−	8.6747	47.3519	0.106	MetaG
	Greifen	SRR11848431	−	8.6747	47.3519	0.106	MetaG
	Greifen	SRR11848394	−	8.6747	47.3519	0.106	MetaG
Czech Republic	Rimov	ERR3761221	+	14.487639	48.846361	0.319	MetaG
	Rimov	ERR3761194	−	14.487639	48.846361	0.319	MetaG
	Rimov	ERR3761195	−	14.487639	48.846361	0.319	MetaG
	Rimov	ERR3761196	−	14.487639	48.846361	0.319	MetaG
	Rimov	ERR3761197	−	14.487639	48.846361	0.319	MetaG
	Rimov	ERR3761198	−	14.487639	48.846361	0.319	MetaG
	Rimov	ERR3761199	−	14.487639	48.846361	0.319	MetaG
	Rimov	ERR3761200	−	14.487639	48.846361	0.319	MetaG
	Rimov	ERR3761201	−	14.487639	48.846361	0.319	MetaG
	Rimov	ERR3761202	−	14.487639	48.846361	0.319	MetaG
	Rimov	ERR3761203	−	14.487639	48.846361	0.319	MetaG
	Rimov	ERR3761204	−	14.487639	48.846361	0.319	MetaG
	Rimov	ERR3761205	−	14.487639	48.846361	0.319	MetaG
	Rimov	ERR3761206	−	14.487639	48.846361	0.319	MetaG
	Rimov	ERR3761207	−	14.487639	48.846361	0.319	MetaG
	Rimov	ERR3761208	−	14.487639	48.846361	0.319	MetaG
	Rimov	ERR3761209	−	14.487639	48.846361	0.319	MetaG
	Rimov	ERR3761210	−	14.487639	48.846361	0.319	MetaG
	Rimov	ERR3761211	−	14.487639	48.846361	0.319	MetaG
	Rimov	ERR3761212	−	14.487639	48.846361	0.319	MetaG
	Rimov	ERR3761213	−	14.487639	48.846361	0.319	MetaG
	Rimov	ERR3761214	−	14.487639	48.846361	0.319	MetaG
	Rimov	ERR3761215	−	14.487639	48.846361	0.319	MetaG
	Rimov	ERR3761216	−	14.487639	48.846361	0.319	MetaG
	Rimov	ERR3761217	−	14.487639	48.846361	0.319	MetaG
	Rimov	ERR3761218	−	14.487639	48.846361	0.319	MetaG
	Rimov	ERR3761219	−	14.487639	48.846361	0.319	MetaG
	Rimov	ERR3761220	−	14.487639	48.846361	0.319	MetaG
	Rimov	ERR3761222	−	14.487639	48.846361	0.319	MetaG
	Rimov	ERR3761223	−	14.487639	48.846361	0.319	MetaG
	Rimov	ERR3761224	−	14.487639	48.846361	0.319	MetaG
	Rimov	ERR3761225	−	14.487639	48.846361	0.319	MetaG
	Rimov	ERR3761226	−	14.487639	48.846361	0.319	MetaG
	Rimov	ERR3761227	−	14.487639	48.846361	0.319	MetaG
	Rimov	ERR3761228	−	14.487639	48.846361	0.319	MetaG
	Rimov	ERR3761229	−	14.487639	48.846361	0.319	MetaG
	Rimov	ERR3761230	−	14.487639	48.846361	0.319	MetaG
	Rimov	ERR3761231	−	14.487639	48.846361	0.319	MetaG
	Jiřická	ERR3761232	−	14.676594	48.616034	0.063	MetaG
	Jiřická	ERR3761233	−	14.676594	48.616034	0.063	MetaG
	Jiřická	ERR3761234	−	14.676594	48.616034	0.063	MetaG
	Jiřická	ERR3761235	−	14.676594	48.616034	0.063	MetaG
	Jiřická	ERR3761236	−	14.676594	48.616034	0.063	MetaG
	Jiřická	ERR3761237	−	14.676594	48.616034	0.063	MetaG
	Jiřická	ERR3761238	−	14.676594	48.616034	0.063	MetaG
	Jiřická	ERR3761239	−	14.676594	48.616034	0.063	MetaG
	Jiřická	ERR3761240	−	14.676594	48.616034	0.063	MetaG
	Jiřická	ERR3761241	−	14.676594	48.616034	0.063	MetaG
Germany	Sankelmark	SRR10607537	−	9.43333333	54.710833	0.267	MetaG
	Roxheimer Altrhein	SRR10607543	−	8.369489	49.57846	0.845	MetaG
	Meerfelder Maar	SRR10607550	−	6.76335	50.100403	0.146	MetaG
	Großes Heiliges Meer	SRR10607568	−	7.63277777	52.348889	0.510	MetaG
	Wilder	SRR10607570	−	10.20027778	49.967222	0.644	MetaG
	Lütschestausee	SRR10607571	−	10.75666667	50.733611	0.060	MetaG
	Kummerower	SRR10607573	−	12.81277778	53.793611	0.838	MetaG
Italy	Viverone	SRR10607546	+	8.048519	45.4175	0.537	MetaG
	Gioveretto	SRR10607541	−	10.71755	46.491917	0.057	MetaG
	Lugano	SRR10607544	−	9.05234	46.023797	0.104	MetaG
	Sillara	SRR10607547	−	10.07026	44.36448	0.055	MetaG
	Castel San Vincenzo	SRR10607548	−	14.055703	41.647629	0.118	MetaG
	Campotosto	SRR10607549	−	13.370268	42.52795	0.055	MetaG
France	Ouillette	SRR10607545	−	6.99512	45.429787	0.055	MetaG
	Retenue de Pincemaille	SRR10607551	+	0.221633	47.462361	0.587	MetaG
	Cap-de-Long	SRR10607553	−	0.140444	42.819094	0.058	MetaG
	Matemale	SRR10607555	−	2.111856	42.573792	0.057	MetaG
	Angoustrine-Villeneuve-des-Escaldes	SRR10607556	−	1.962192	42.577056	0.057	MetaG
	L’Homol	SRR10607557	−	4.047225	44.319533	0.499	MetaG
	Réservoir de Panthier	SRR10607558	−	4.631361	47.238072	0.163	MetaG
	Lac du Bouchet	SRR10607569	−	3.792808	44.906383	0.063	MetaG
Spain	Gállego	SRR11430614	−	−0.261183	42.775875	0.058	MetaG
	Tous	SRR5338504	−	−0.65	39.14	0.774	MetaG
	Tous	SRR4198832	−	−0.65	39.14	0.774	MetaG
	Tous	SRR4198666	−	−0.65	39.14	0.774	MetaG
	Amadorio	SRR1173821	−	−0.2663	38.5355	0.835	MetaG
	Embassament d’Utxesa	SRR11430615	+	0.512892	41.497325	0.825	MetaG
	Embalse de Mediano	SRR10607554	−	0.191717	42.323422	0.438	MetaG
	Redon	ERR472738	+	0.7784	42.6411	0.059	MetaG
Poland	Turawskie	SRR10607567	−	18.107183	50.720583	0.712	MetaG
	Piecnickie	SRR10607572	−	16.25416667	53.3425	0.487	MetaG
Finland	Alinen Mustajärvi	ERR4193363	−	25.11388889	61.2080556	0.110	MetaG
	Alinen Mustajärvi	ERR4193366	−	25.11388889	61.2080556	0.110	MetaG
	Alinen Mustajärvi	ERR4195023	−	25.11388889	61.2080556	0.110	MetaG
	Alinen Mustajärvi	ERR4194908	−	25.11388889	61.2080556	0.110	MetaG
	Alinen Mustajärvi	ERR4195937	−	25.11388889	61.2080556	0.110	MetaG
	Keskinen Rajajärvi	ERR4193966	−	25.21555556	61.2161111	0.091	MetaG
	Keskinen Rajajärvi	ERR4195119	−	25.21555556	61.2161111	0.091	MetaG
	Keskinen Rajajärvi	ERR4194057	−	25.21555556	61.2161111	0.091	MetaG
	Keskinen Rajajärvi	ERR4195120	−	25.21555556	61.2161111	0.091	MetaG
	Keskinen Rajajärvi	ERR4195121	−	25.21555556	61.2161111	0.091	MetaG
	Mekkojärvi	ERR4197939	−	25.14222222	61.2308333	0.097	MetaG
	Mekkojärvi	ERR4195215	−	25.14222222	61.2308333	0.097	MetaG
	Mekkojärvi	ERR4195061	−	25.14222222	61.2308333	0.097	MetaG
	Mekkojärvi	ERR4194702	−	25.14222222	61.2308333	0.097	MetaG
	Valkea Kotinen	ERR4194718	−	25.06305556	61.2422222	0.108	MetaG
	Valkea Kotinen	ERR4194719	−	25.06305556	61.2422222	0.108	MetaG
	Valkea Kotinen	ERR4195071	−	25.06305556	61.2422222	0.108	MetaG
	Valkea Kotinen	ERR4195217	−	25.06305556	61.2422222	0.108	MetaG
	Ylinen Rajajärvi	ERR4195070	−	25.2125	61.2180556	0.091	MetaG
	Ylinen Rajajärvi	ERR4195072	−	25.2125	61.2180556	0.091	MetaG
	Ylinen Rajajärvi	ERR4194720	−	25.2125	61.2180556	0.091	MetaG
	Ylinen Rajajärvi	ERR4195067	−	25.2125	61.2180556	0.091	MetaG
Sweden	Bengtgölen	ERR4194562	−	16.19083333	58.6961111	0.699	MetaG
	Erken	ERR4193663	−	18.64194444	59.8363889	0.422	MetaG
	Erken	ERR4193664	−	18.64194444	59.8363889	0.422	MetaG
	Erken	ERR4195036	−	18.64194444	59.8363889	0.422	MetaG
	Erken	ERR4195118	−	18.64194444	59.8363889	0.422	MetaG
	Erken	ERR4193931	−	18.64194444	59.8363889	0.422	MetaG
	Erken	ERR4195041	−	18.64194444	59.8363889	0.422	MetaG
	Erken	ERR4193668	−	18.64194444	59.8363889	0.422	MetaG
	Erken	ERR4195029	−	18.64194444	59.8363889	0.422	MetaG
	Erken	ERR4193667	−	18.64194444	59.8363889	0.422	MetaG
	Erken	ERR4195032	−	18.64194444	59.8363889	0.422	MetaG
	Erken	ERR4210440	−	18.64194444	59.8363889	0.422	MetaG
	Erken	ERR4194707	−	18.64194444	59.8363889	0.422	MetaG
	Fyrsån	ERR4195073	−	18.50611111	59.7975	0.705	MetaG
	Glimmingen	ERR4195045	−	15.57222222	57.9336111	0.756	MetaG
	Långsjön	ERR4195062	−	17.56361111	60.0386111	0.846	MetaG
	Lillsjon	ERR4195883	−	16.14361111	59.6422222	0.667	MetaG
	Lomtjärnan	ERR4195094	−	14.45888889	63.3491667	0.276	MetaG
	Lomtjärnan	ERR4195093	−	14.45888889	63.3491667	0.276	MetaG
	Lomtjärnan	ERR4193365	−	14.45888889	63.3491667	0.276	MetaG
	Lomtjärnan	ERR4195107	−	14.45888889	63.3491667	0.276	MetaG
	Lomtjärnan	ERR4195025	−	14.45888889	63.3491667	0.276	MetaG
	Lomtjärnan	ERR4193666	−	14.45888889	63.3491667	0.276	MetaG
	Lomtjärnan	ERR4193665	−	14.45888889	63.3491667	0.276	MetaG
	Lomtjärnan	ERR4193370	−	14.45888889	63.3491667	0.276	MetaG
	Lomtjärnan	ERR4195024	−	14.45888889	63.3491667	0.276	MetaG
	Lomtjärnan	ERR4193652	−	14.45888889	63.3491667	0.276	MetaG
	Lomtjärnan	ERR4195111	−	14.45888889	63.3491667	0.276	MetaG
	Lomtjärnan	ERR4195039	−	14.45888889	63.3491667	0.276	MetaG
	Lomtjärnan	ERR4195923	−	14.45888889	63.3491667	0.276	MetaG
	Lomtjärnan	ERR4195122	−	14.45888889	63.3491667	0.276	MetaG
	Lomtjärnan	ERR4194175	−	14.45888889	63.3491667	0.276	MetaG
	Lomtjärnan	ERR4194085	−	14.45888889	63.3491667	0.276	MetaG
	Lomtjärnan	ERR4194104	−	14.45888889	63.3491667	0.276	MetaG
	Lotsjön	ERR4195064	−	17.93888889	59.8622222	0.831	MetaG
	Malstasjön	ERR4194708	−	18.64277778	59.7688889	0.610	MetaG
	Parsen	ERR4195046	−	16.20388889	58.3402778	0.843	MetaG
	Plåten	ERR4194710	−	18.5425	59.8625	0.444	MetaG
	Stortoveln	ERR4195051	−	15.55166667	57.9330556	0.756	MetaG
	Ymsen	na	−	58.707327	14.003391	0.574	Field
	Hornborgasjön	na	−	58.3231599	13.5284973	0.728	Field
	Tåkern	na	−	58.332558	14.8204108	0.866	Field
	Boren	na	−	58.56756	15.0906993	0.802	Field
	Mälaren	na	−	59.452594	16.7308041	0.854	Field
	Fjällfotasjön	na	−	55.5245925	13.2942426	0.180	Field
	Ringsjön	na	−	55.9089848	13.4376851	0.266	Field
	Vombsjön	na	−	55.6988186	13.5549667	0.301	Field
	Finjasjön	na	−	56.1295179	13.6875359	0.544	Field
	Yddingesjön	na	−	55.5513814	13.2602208	0.221	Field
	Sjön	na	−	55.7101586	13.2083502	0.222	Field

Accession number for publicly available metagenomic datasets from the National Center for Biotechnology Information (https://www.ncbi.nlm.nih.gov/) used for in-silico screening. Probability of occurrence column is taken from Species Distribution Models (SDMs) output (Meriggi et al., 2022), corresponding to high (>0.5) or low (<0.5) probability of occurrence of the invasive *Raphidiopsis raciborskii*.

**Fig. 1:**
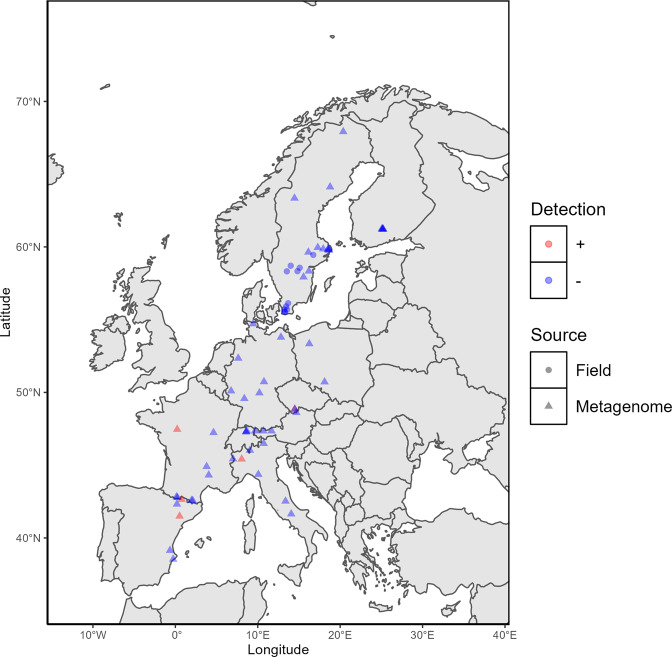
Detection of *Raphidiopsis raciborskii* in screened samples and metagenomes. Detected presence (+) and absence (−) of the invasive cyanobacterium *Raphidiopsis raciborskii* in freshwater lakes and reservoirs across Europe based on field (only Sweden) and in-silico screening of environmental DNA using publicly available metagenomic datasets.

While SDMs are valuable tools for predicting potential invasion sites and to guide management efforts, many uncertainties remain. One of the most important limitations when constructing the SDMs was the general lack of relevant environmental variables for predicting the range expansion of the invasive species. Reports of presence are not usually accompanied by detailed environmental metadata, such as temperature and nutrients, that are known to be important for phytoplankton [9], and knowledge of interactions with native species in invaded areas is largely lacking. This suggests that frequent monitoring and open access to additional biotic and abiotic data connected to the presence of the target species in already invaded areas are necessary for developing high grid resolution and more accurate models to predict the likelihood of invasion into new aquatic environments.

## Supplementary information

Supplementary Material

## Data Availability

All datasets used in this article are publicly available. The accession numbers are mentioned in the article (Table 1) and Supplementary Material.
