# Supplementary material for: Challenges in supplying empirical proof for predictions derived from Species Distribution Models (SDMs): the case of an invasive cyanobacterium
Source: ISME Commun. 2023 Jun 6;3:56. doi: 10.1038/s43705-023-00264-2 (PMC10244341; doi:10.1038/s43705-023-00264-2)
Supplement: Supplementary file 1 — Supplementary Material [file 43705_2023_264_MOESM1_ESM.docx]

# **Supplementary material**

**Challenges in supplying empirical proof for predictions derived from Species Distribution Models (SDMs): the case of an invasive cyanobacterium**

Carlotta Meriggi ^1*^, Maliheh Mehrshad ^1^, Richard K. Johnson ^1^, Ane T. Laugen ^2^, Stina Drakare ^1^

*^1^ Department of Aquatic Sciences and Assessment, Swedish University of Agricultural Sciences, Uppsala, Sweden*

^2^ *Department of Natural Sciences, Centre for Coastal Research, University of Agder, Kristiansand, Norway*

* Corresponding author: [carlotta.meriggi@slu.se](mailto:carlotta.meriggi@slu.se)

**S.1. Methods and results**

**S.1.1. Sample collection:** Based on SDMs prediction (1) six lakes Ymsen, Hornborgasjön, Tåkern, Boren, Mälaren and Finjasjön with high probability of occurrence (>0.5) and five lakes Fjällfotasjön, Ringsjön, Vombsjön, Yddigasjön and Sjön with low probability of occurrence (<0.5) for the invasive cyanobacterium *Raphidiopsis raciborskii* were sampled in August 2020 (Table S1). We selected eutrophic shallow lakes (Tåkern, Hornborgasjön, Fjällfotasjön and Sjön do not surpass 3 m of depth) known to be waterfowl resting lakes, since birds may act as vectors for dispersal. Such lakes are usually warm and nutrient rich, favoring cyanobacterial blooms. In each lake, five replicates for both water and sediment were sampled (for Lake Sjön only one replicate was collected) along the lake’s perimeter, as many of the lakes were bird reserves, not allowing boats during summer. For water samples, up to 8L of water was passed through a phytoplankton net (20-25 µm mesh size) to be able to concentrate large volumes of lake water to get a thick sample of phytoplankton. The concentrated samples were stored at 4 °C in 0.5 L bottles in the field. Concentrated phytoplankton samples were also collected in glass bottles and preserved with acid Lugol’s solution for further microscopic analysis. When back in the laboratory, from the concentrated sample, algal biomass was collected by filtering it through GF/F filters (Whatman, UK) with a mesh size of 0.7 µm. Filters were stored at -80°C until extraction of DNA. Sediment samples were collected at the same location as water samples by removing surficial sediment using a grabbing device. Sediment samples were transferred to 50 ml falcon tubes and stored at -20°C until processing.

**Table S1.** Sampling information and abiotic conditions for the 11 sampled lakes in Sweden.

| Lake | Sampling site at each sampling location | Date | WaterTemp  (°C) | Air  Temp  (°C) | Water depth at the point of sediment sampling (cm) | Weather conditions during sampling |
| --- | --- | --- | --- | --- | --- | --- |
| Ymsen | Beach | 2020-08-05 | 17.6 | 15 | 50 | cloudy, windy, rainy |
| Ymsen | Private beach1 | 2020-08-05 | 17.6 | 15 | 50 | cloudy, windy, rainy |
| Ymsen | Private beach2 | 2020-08-05 | 16.2 | 14.8 | 50 | cloudy, windy, rainy |
| Ymsen | Private beach3 | 2020-08-05 | 18.6 | 16.1 | 36 | cloudy, windy, rainy |
| Ymsen | Private beach4 | 2020-08-05 | 17.8 | 16.8 | 52 | cloudy, windy, rainy |
| Hornborgasjön | Beach1 | 2020-08-06 | 19.2 | 23 | 70 | sunny, warm, wind |
| Hornborgasjön | Beach2 | 2020-08-06 | 21.7 | 25.5 | 40 | sunny, warm, wind |
| Hornborgasjön | Beach3 | 2020-08-06 | 23.8 | 25.7 | 50 | sunny, warm, wind |
| Hornborgasjön | Nature reserve | 2020-08-06 | 22.1 | 25 | 35 | sunny, warm, wind |
| Hornborgasjön | Beach | 2020-08-06 | 25.1 | 25 | 50 | sunny, warm, wind |
| Tåkern | Nature reserve | 2020-08-07 | 21.7 | 25 | 51 | sunny, clear |
| Tåkern | Beach1 | 2020-08-07 | 23.9 | 26.5 | 40 | sunny, clear |
| Tåkern | Beach2 | 2020-08-07 | 25.5 | 26 | 40 | sunny, clear |
| Tåkern | Beach3 | 2020-08-07 | 25.1 | 26.7 | 60 | sunny, clear |
| Tåkern | Private property | 2020-08-07 | 24.5 | 27 | 100 | sunny, clear |
| Boren | Private property1 | 2020-08-08 | 20 | 21.4 | 95 | sunny, windy |
| Boren | Private property2 | 2020-08-08 | 20.7 | 22.7 | 62 | sunny, windy |
| Boren | Beach1 | 2020-08-08 | 21 | 25 | 50 | sunny, windy |
| Boren | Private property3 | 2020-08-08 | 22 | 22 | 72 | sunny, windy |
| Boren | Beach2 | 2020-08-08 | 20.4 | 24.8 | 50 | sunny, windy |
| Mälaren | Private property1 | 2020-08-09 | 22.7 | 25.3 | 80 | sunny, clear |
| Mälaren | Private property2 | 2020-08-09 | 23 | 22.6 | 118 | sunny, clear |
| Mälaren | Beach1 | 2020-08-09 | 24 | 26 | 180 | sunny, clear |
| Mälaren | Private property3 | 2020-08-09 | 23.6 | 25.7 | 60 | sunny, clear |
| Mälaren | Beach2 | 2020-08-09 | 23 | 25.3 | 170 | sunny, clear |
| Fjällfotasjön | Beach1 | 2020-08-18 | 23 | 23.5 | 100 | sunny, calm |
| Fjällfotasjön | Beach2 | 2020-08-18 | 23.4 | 26 | 65 | sunny, warm |
| Fjällfotasjön | Bridge | 2020-08-18 | 24.2 | 26.2 | 112 | sunny, warm |
| Fjällfotasjön | Private property | 2020-08-18 | 26 | 26.7 | 70 | sunny, warm |
| Fjällfotasjön | Beach3 | 2020-08-18 | 26 | 26.2 | 100 | sunny, warm |
| Ringsjön | Beach1 | 2020-08-19 | 22.6 | 18.6 | 45 | fog |
| Ringsjön | Beach2 | 2020-08-19 | 22.6 | 22 | 40 | fog, sun |
| Ringsjön | Beach3 | 2020-08-19 | 22.4 | 21.6 | 50 | sun |
| Ringsjön | Beach4 | 2020-08-19 | 22 | 23.3 | 60 | cloudy |
| Ringsjön | Beach5 | 2020-08-19 | 22 | 21.4 | 45 | cloudy, thunder |
| Vombsjön | Beach1 | 2020-08-19 | 24.3 | 20 | 50 | cloudy, slightly rain |
| Vombsjön | Beach2 | 2020-08-19 | 24 | 19 | 50 | cloudy, slightly rain |
| Vombsjön | Beach3 | 2020-08-19 | 22.4 | 20 | 60 | cloudy, slightly rain |
| Vombsjön | Private property | 2020-08-19 | 23.6 | 20.7 | 60 | cloudy, slightly rain |
| Vombsjön | Beach4 | 2020-08-19 | 24 | 23.1 | 60 | sun |
| Finjasjön | Beach1 | 2020-08-20 | 22.4 | 19.2 | 97 | fog |
| Finjasjön | Beach2 | 2020-08-20 | 22 | 20.7 | 60 | fog |
| Finjasjön | Beach3 | 2020-08-20 | 22 | 25.2 | 70 | fog |
| Finjasjön | Beach4 | 2020-08-20 | 21.8 | 24.9 | 40 | fog |
| Finjasjön | Beach5 | 2020-08-20 | 23 | 24.9 | 50 | fog |
| Yddingasjön | Private property1 | 2020-08-21 | 22.2 | 21.2 | 50 | cloudy |
| Yddingasjön | Beach | 2020-08-21 | 22.3 | 23.6 | 75 | rain |
| Yddingasjön | Private property2 | 2020-08-21 | 21.6 | 25.5 | 96 | rain |
| Yddingasjön | Private property3 | 2020-08-21 | 22.8 | 24 | 140 | rain |
| Yddingasjön | Private property4 | 2020-08-21 | 22.4 | 20.7 | 95 | rain |
| Sjön | City park, Lund | 2020-08-21 | 20.7 | 21 | 21 | rain |

**S.1.2. Microscopic identification:** The phytoplankton samples were preserved with acid Lugol’s solution and cyanobacteria were identified using an inverted light microscope and a modified Utermöhl technique commonly used in Scandinavia (Olrik et al., 1989). The microscopic search after *R. raciborskii* was negative.

**S.1.3. DNA extraction, PCR amplification:** DNeasy PowerSoil Pro Kits (Qiagen) was used for extracting DNA from filters and sediment with an adjusted protocol regarding GF/F filters (water samples) according to the manufacturer's instructions (Qiagen):

Glass fiber filter (GF/F) membranes are highly absorbent and will retain most if not all of the lysis buffer, regardless of membrane size (25 mm, 47 mm). In order to recover the supernatant after the mechanical lysis step, the membrane must be placed onto a column that will retain the membrane but allow the supernatant to be centrifuged out for use in subsequent steps. We removed the plunger from a 5 ml syringe barrel and discarded it. After the mechanical lysis we transferred the content of the lysis tube into the syringe barrel and placed the barrel into a 15 ml tube and centrifuged for 3 minutes at 4 000 x g. The supernatant accumulated in the 15 ml tube while the membrane was retained in the syringe barrel. After lysis and recover of supernatant step the standard protocol from the DNeasy PowerSoil Pro Kits (Qiagen) was followed.

The extracted DNA was used for PCR amplification of the gene rpoC1 with *R. raciborskii* specific primers cyl2/cyl4 (2) and cyl4F/cyl4R (3). All PCR reactions were carried out in the volume of 50 μL by using the DreamTaq PCR Master Mix (Thermo Scientific, USA). The amplification program was as follows: initial DNA denaturation for 3 min at 98 °C; 30 amplification cycles (one cycle was 30 s at 98 °C, 30 s at 55 °C and 30 s at 72 °C); and a final elongation for 2 min at 72 °C. PCR products were separated by electrophoresis on 1.5% agarose gel, stained with Gelred, and visualized under UV illumination. The size of the amplified DNA fragments was determined using the FastRuler Low Range DNA Ladder (Thermo Scientific, USA) ranging from 100 to 1000 bp molecular weight.

**S.1.4. Metagenomes analysis:** A total of 153 publicly available metagenomic datasets were downloaded from NCBI using parallelfastq-dump (<https://github.com/rvalieris/parallel-fastq-dump>). The downloaded metagenomes were quality checked using the bbduk.sh script (<https://sourceforge.net/projects/bbmap/>). After the quality control, the seqtk tool (<https://github.com/lh3/seqtk>) was used for processing sequences from FastQ to Fasta format and selecting 10 million random reads. SSU-align tool (4) was then used for identifying, aligning, masking and visualizing bacterial 16S rRNA related reads. It enabled us to generate large-scale alignments of up to millions of SSU rRNA sequences based on the conserved secondary structure and sequence of SSU rRNA. The taxonomy of the extracted 16S rRNA reads was assigned using BLAST (5) against Silva SSU 138.1 (6) as reads assigned to invasive cyanobacterium *Raphidiopsis raciborskii* were detected.


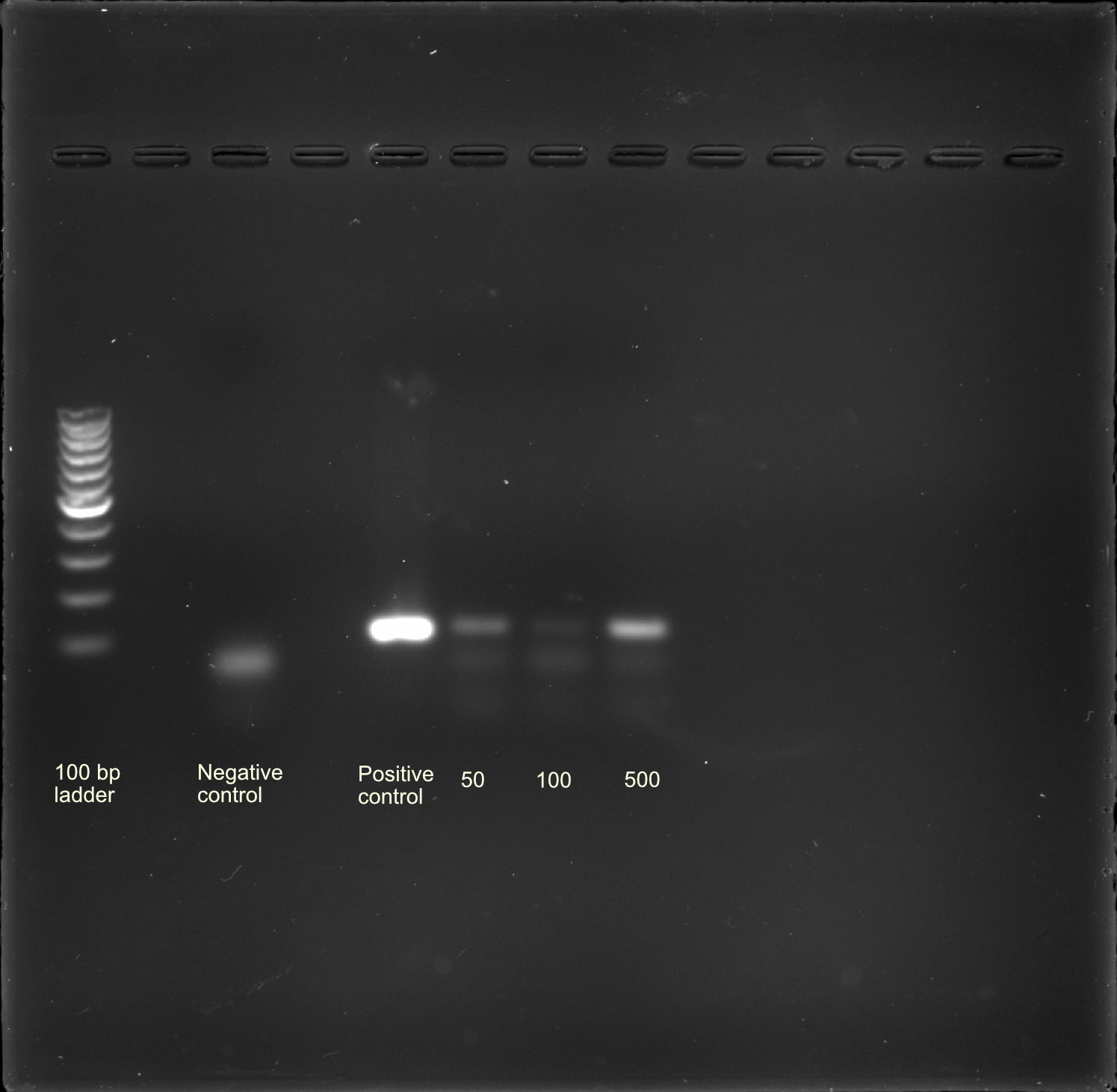

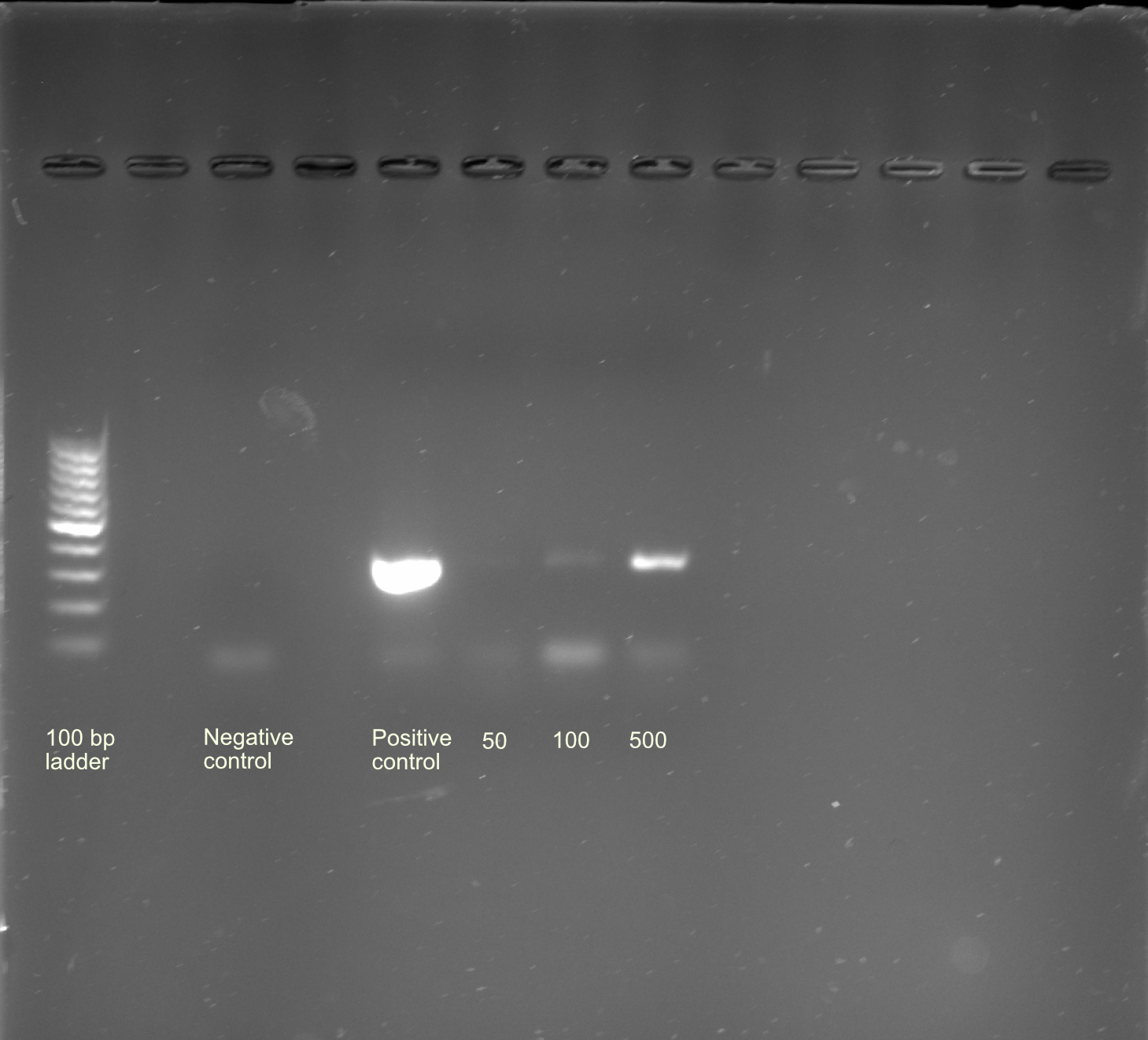


**Figure S1.** PCR detection limits for 50, 100, and 500 filaments of the reference culture of *R. raciborskii*. Left) The chloroplast (rpoC1) gene was targeted and amplified with *R. raciborskii* specific primers cyl4F/cyl4R (110 bp). Right) The chloroplast (rpoC1) gene was targeted and amplified with *R. raciborskii* specific primers cyl2/cyl4 (308 bp).

**Reference**

1. Meriggi C, Drakare S, Polaina Lacambra E, Johnson RK, Laugen AT. Species distribution models as a tool for early detection of the invasive *Raphidiopsis raciborskii* in European lakes. Harmful Algae. 2022 Mar 1;113.

2. Wilson KM, Schembri MA, Baker PD, Saint CP. Molecular characterization of the toxic cyanobacterium *Cylindrospermopsis raciborskii* and design of a species-specific PCR. Appl Environ Microbiol. 2000;66(1):332–8.

3. Lei L, Lei M, Lu Y, Peng L, Han BP. Development of real-time PCR for quantification of *Cylindrospermopsis raciborskii* cells and potential cylindrospermopsin-producing genotypes in subtropicalreservoirs of southern China. J Appl Phycol. 2019;31(6):3749–58.

4. Nawrocki E. Structural RNA Homology Search and Alignment Using Structural RNA Homology Search and Alignment Using Covariance Models Covariance Models. 2009 [cited 2022 Dec 7]; Available from: https://openscholarship.wustl.edu/etd/256

5. Wheeler DL, Barrett T, Benson DA, Bryant SH, Canese K, Chetvernin V, et al. Database resources of the National Center for Biotechnology Information. Nucleic Acids Res. 2007;35(SUPPL. 1):5–12.

6. Quast C, Pruesse E, Yilmaz P, Gerken J, Schweer T, Yarza P, et al. The SILVA ribosomal RNA gene database project: Improved data processing and web-based tools. Nucleic Acids Res. 2013;41(D1):590–6.

**Websites**

BBMap. SourceForge. Retrieved 2021, from https://sourceforge.net/projects/bbmap/

Lh3/SEQTK: Toolkit for processing sequences in FASTA/Q Formats. GitHub. Retrieved 2021, from <https://github.com/lh3/seqtk>

Rvalieris/parallel-FASTQ-dump: Parallel FASTQ-Dump Wrapper. GitHub. Retrieved 2021, from https://github.com/rvalieris/parallel-fastq-dump
